# UV index-based model for predicting synthesis of (pre-)vitamin D3 in the mediterranean basin

**DOI:** 10.1038/s41598-024-54188-5

**Published:** 2024-02-12

**Authors:** Mehmet Ali Kallioğlu, Ashutosh Sharma, Ayşan Kallioğlu, Sunil Kumar, Rohit Khargotra, Tej Singh

**Affiliations:** 1 Besiri OSB Vocational School, Batman University, 72060 Batman, Turkey; 2 College of Science and Engineering, James Cook University, Townsville, QLD 4810 Australia; 3 Department of Neurology, Faculty of Medicine, Cigli Research and Training Hospital, Izmir Bakırçay University, 8780 Çiğli - İzmir, Turkey; 4 Department of Nanotechnology and Advanced Materials Engineering and HMC, Sejong University, Seoul, 05006 South Korea; 5 Institute of Materials Engineering, Faculty of Engineering, Pannonia University, Veszprem, 8200 Hungary; 6 Sustainability Solutions Research Lab, University of Pannonia, Egyetem u. 10., Veszprém, 8200 Hungary; 7 Savaria Institute of Technology, Faculty of Informatics, ELTE Eötvös Loránd University, Budapest, Budapest, 1117 Hungary

**Keywords:** UV radiation, Vitamin D_3_, Solar zenith angle, Simulation, Solar energy, Energy science and technology, Engineering

## Abstract

The importance of solar radiation for the body’s ability to synthesize Vitamin D_3_ is well documented, yet the precise amount of sun exposure required to avoid Vitamin D insufficiency is less clear. To address this knowledge gap, this study sought to utilize the sun in a suitable period at the optimum dose by utilizing numerical simulations to determine the amount of Vitamin D_3_ synthesis in the skin according to season, time of day, and geographical location in Turkey. The study was carried out in three stages; in the first stage, daily, monthly, and annual values were determined in cases where the solar zenith angle has the active UV-B wavelength. The second stage determined the level of Vitamin D that can be synthesized in all skin types at 25% solar radiation exposure. In the third stage, the sun exposure time required for 1000 International Units (IU) for all skin types was calculated. According to the analysis, the yearly period of active synthesis of D_3_ on Earth lasts from the beginning of March to the third week of October. During the day, it is between 10:00 and 16:00. For 1000 IU/day, the average annual estimated times (minutes) are 5.05 for Type I, 6.3 for Type II, 7.6 for Type III, 11.35 for Type IV, 15.15 for Type V, and 25.25 for Type VI. The results of this paper will impact awareness for academic-medical users.

## Introduction

According to Hippocrates, the sun was beneficial for treating various diseases. However, the industrial revolution of the nineteenth century led to a pandemic of rickets in children and osteomalacia in adults due to the mass migration from rural to urban areas ^1,2^. In the early twentieth century, when the relationship between UV rays and rickets was investigated, it was seen that children recovered with the synthesis of vitamin D_3_, which increased in the body as they were exposed to UVB radiation^3,4^. Thus, solar radiation was thought to be a panacea for living things. However, in a study on mice exposed to ultraviolet (UV) rays, Deoxyribose Nucleic acid in their skin (DNA) mutations, leading to the development of tumors and cancer ^5^. In 1937, Peller and Stephenson found that skin cancer rates were eight times higher than expected in the US Navy due to increased exposure to solar UV radiation. This pioneering study has greatly influenced our understanding of sun exposure risks and the need for protective measures ^6^. As a result of all these situations, it is necessary to utilize the sun neither too little nor too much, that is, at a suitable time, in sufficient doses (optimum).

Sun exposure has benefits in preventing and treating skin conditions such as psoriasis and eczema, as well as seasonal affective disorder and contamination from SARS-CoV-2^7^. Recent years have discovered several novel links between illnesses and sun radiation. Regular sun exposure may help prevent diseases such as colorectal, breast, and prostate cancer, non-lymphoma Hodgkin's (NHL), multiple sclerosis (MS), hypertension, and diabetes mellitus (DM), according to studies that have offered growing amounts of observational and experimental data. This has been attributed to increased vitamin D synthesis in the individual^8^. COVID-19, caused by SARS-CoV-2, is a top global pandemic with severe respiratory symptoms^9^. In the fight against the pandemic, the positive effect of vitamin D supplementation in reducing the risk of respiratory infections has been reported in many studies^10–19^. The clinical studies have shown that vitamin D_2_ and D_3_ supplements can greatly decrease the risk of COVID-19 infection and mortality. Specifically, vitamin D_3_ supplementation for 30 days has been proven to significantly reduce both infection and death from the virus, implying its potential use in improving immunity. These findings should be further investigated and taken into account for pandemic control^20^. The National Academy of Sciences America has noted the advantageous effect of UV light in lowering COVID-19 development rates. It was shown that local COVID-19 growth rates are decreased by 0.09 (± 0.04, *P* = 0.01) for every 1 kJ m^−2^ h^−1^ rise in local UV^21^. Vitamin D affects many tissues and systems in the human body^22^. About 3% of the human genome is controlled by 1.25(OH)_2_, the active form of vitamin D_3_^23^. Especially at the beginning of the twenty-first century, more than 1 billion people worldwide have insufficient exposure to direct sunlight. The highest-risk groups susceptible to diseases related to vitamin D deficiency are pregnant, obese, children, and adults with dark skin color^24,25^. The reasons for this deficiency include decreased synthesis in the skin (aging, skin pigmentation, UVB amount-effectiveness, altitude, air pollution, time of day, cloud cover, use of protective cream), decreased bioavailability (fat malabsorption, obesity), drugs that increase catabolism (anticonvulsants, antifungal, antituberculosis, anti-retroviral and glucocorticoids), defective 25-hydroxylation (liver diseases), increased urinary (nephrotic syndrome), defective 1-alpha 25-hydroxylation (renal failure, hypoparathyroidism, 1-alpha-hydroxylase deficiency)^26–28^.

A global public health issue is insufficient levels of vitamin D, with an estimated 10% of Europeans having a severe deficiency of less than 12 ng/mL. In Northern Europe, around 20% have a deficiency of less than 20 ng/mL, while in other European regions and the Middle East, the percentages are higher (30–60% and 80%, respectively)^29^. In the selected cities in this study where vitamin D levels were analyzed, according to a study conducted in Istanbul-Turkey 41°N locations, the insufficiency rate in women aged 14–44 years was found to be between 44 and 100%^30^. Antalya-Turkey 37° N operating room staff vitamin D levels were examined, and it was found that 22% had vitamin D levels below 10 ng/ml, 63% had vitamin D levels between 11 and 20 ng/ml, and 15% had vitamin D levels above 20 ng/ml^31^. In addition, a study on subjects from different ethnic groups with skin type VI living in the African continent, especially at low latitudes (2–4°S' of the Equator), such as Tanzania, revealed interesting results. Their cultural style includes low fish consumption, moderate clothing, and spending most of the day outdoors. These assessments found that the average 25(OH)D levels of traditionally living African subjects exceeded 100 nmol/L (> 40 ng/mL). This situation can be associated with various factors. The first factor is intense exposure to tropical sunlight. The second factor is Paleolithic genetic inheritance and primitive lifestyle. The genetic makeup of African subjects may have adapted to higher vitamin D_3_ synthesis. However, larger studies are needed to generalize these findings, and it is essential to consider individual differences^32,33^.

**Figure 1:**
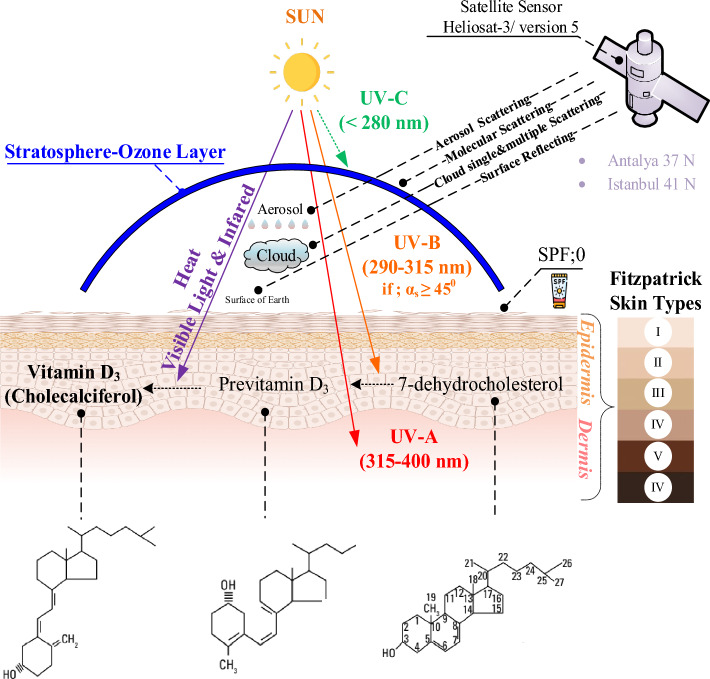
Vitamin D_3_ synthesis steps and UV radiation.

Biologically active ultraviolet (UV) spectra have influenced fundamental photochemical processes that determine not only biological responses, but also biological evolution on the basis of thermodynamic principles. This has played a crucial role in the origin of life on Earth, as simple organic molecules utilized UV energy to convert it into high-energy chemical bonds. This, especially in the early stages of life’s development (biogenesis), provided cells with an additional source of energy. For instance, vitamin D_2_ is produced from ergosterol and vitamin D_3_ is produced from 7-dehydrocholesterol through UVB-induced carbon–carbon bond cleavage^34^. Vitamin D is fat-soluble and can be taken as ergocalciferol (D_2_) and cholecalciferol (D_3_). If one uses the sun as a source of vitamin D, it is synthesized from 7- dehydrocholesterol (7-DHC), which starts when your skin is exposed to ultraviolet. In this synthesis, it is converted into pre-vitamin D_3_ by exposure to ultraviolet B (UVB) radiation and into vitamin D_3_ by thermal photoisomerization (≥ 25 °C)^35^. It is assumed that the B ring in the 7-DHK molecule can be opened with the energy of photons in the range of 282–310 nm corresponding to UVB radiation (18 mJ/cm^2^)^36^. Vitamin D made in the skin or obtained from food is not biologically active. Vitamin D_3_ entering the bloodstream is a molecule first taken up in the liver as a hormone precursor to calcidiol [25(OH)D_3_]^37^. Furthermore, it is metabolized in the kidneys by the enzyme one alpha-hydroxylase to the biologically active form calcitriol [1.25(OH)_2_D_3_]^38,39^. The body must have an optimal level of 25D_3_ stored in the liver. This is because these stores increase the body's ability to use its vitamin D capacity, as shown in Fig. 1. Additionally, recent studies have shown that there are differences between UVB-induced vitamin D signalling and oral vitamin D administration. That is, it shows that UVB, which has numerous biological functions independent of calcium and bone metabolism of vitamin D, its photoproducts and metabolites, can produce numerous molecules that can regulate local and global homeostasis^40^. The sun is an indispensable energy source for living things on earth and has beneficial and harmful effects.

Components of solar radiation are categorized into three parts in the electromagnetic spectrum: 3% UV (100–400 nm), 53% visible (400–700 nm), and 44% IR (700 nm-1 mm) radiation. UV radiation, which ranges from 100 to 400 nm, is divided into four components: UVA, UVB, UVC, and vacuum-UV. UV Index values are measured either through ground-based spectrometers or satellite-based data. This index was first measured in 1992 in Canada and is primarily based on constant factors such as albedo, altitude, and aerosol amount. This model has limitations in areas with low ozone levels and certain zenith angles^41^. While local measurements are the most exact, only a limited amount of ground-based measurements are available due to the restricted accessibility to these instruments. Subsequently, studies have been conducted to estimate UV radiation in other geographies using satellite and ground-based techniques^42–44^.

As a result of the effect of radiation on the earth's surface, the UV index plays a key role in vitamin D synthesis. It may also contribute to cancer formation as a result of high-dose exposure. The UV index (UVI) is a globally recognized measure of UVR intensity developed by the world health organization and an indicator of the likelihood of sunburn^45^. UV-A and UV-B rays reach the Earth, while UV-C is filtered by the ozone layer. UV-A is responsible for 95% of UV light reaching the surface, causing photo aging and allergic reactions. UV-B makes up 5% and can be partially filtered by the ozone layer, but still causes skin damage and can initiate vitamin D synthesis. UV-C, the shortest and most powerful wavelength, is absorbed by the stratosphere and used for sterilization. Its intense intervals are 10:00–16:00 during summer months, and it has a strong carcinogenic effect due to its absorption by DNA, RNA, and proteins. The highest antiseptic effect is found at 205–280 nm, with maximum sensitivity at 265 nm^46–50^. The efficacy of radiation at different wavelengths is Human Erythema (ery) 250–400 nm^51^, Previtamin D_3_ production (Vit-D_3_) 252–330 nm^52^ and photocarcinogenesis (nonmelanoma skin cancers-NMC) 250–400 nm^53^.

Another vital factor in vitamin D synthesis is the angle of the sun. Solar zenith angle (SZA) with solar altitude angle is a whole, and their sum equals 90°. Solar altitude angle value is the environmental factor in vitamin D synthesis. It reaches its maximum value at noon when the sun is at its highest position. Thus, as solar radiation reaches the earth and travels less distance, the wavelength level of the radiation decreases. Solar altitude angle is lowest early in the morning and winter, highest in summer and at the equator. With this angle value of 45° and above, the length of the shadow on a horizontal surface is equal to the height of the person casting the shadow. Thus, UVB radiation is less absorbed by the ozone layer and reaches the ground surface, playing a vital role in the synthesis of active vitamin D_3_^54–56^. Also, the In-Vitro studies stated that there is significant evidence of vitamin D_3_ production at solar altitude angle greater than 45° and that sun exposure is unnecessary at angles smaller than this value. Furthermore, 25(OH)D_3_ peaks occurred in September and troughs in March^43,57, 58^.

As a result of latitudinal-seasonal changes and the rotation of the earth around the sun during the year, the solar zenith angle (SZA) increases in winter and decreases in summer. Also, due to the obliquity of the orbit, the SZA is lower around the equator (0°) during the year and increases as we move towards the poles (90°). The increase in the SZA angle value leads to a decrease in the amount of vertical incoming radiation and thus a decrease in the level of ultraviolet radiation reaching the earth. Serum 25(OH)D levels decrease by approximately 4 nmol/L for each degree increase in Earth's orbital latitude^59^. Many studies are showing a positive correlation between decreasing latitude and synthesized D_3_ levels^60^. Ladizesky et al.^61^ In the study for Ushuaia (55° S) and Buenos Aires (34 °S), Argentina, Vitamin D_3_ production between 10:30 a.m. and 2:30 p.m. was 36.54% higher in the city closer to the equator than in the other. Engelsen et al. (2005) examined the dependence of the extent and duration of vitamin D production in a simulation program. Accordingly, for clear atmospheric conditions, vitamin D is not produced through the skin at some times of the year at 51 degrees and higher latitudes. Furthermore, clouds, aerosols, and ozone completely suppress vitamin D synthesis even at the equator or significantly reduce the synthesis time^62^. Zittermann^63^ In current data from the United States and Europe, serum 25(OH)D levels were higher in healthy subjects living at lower latitudes (r 0.68; *P* 0.01) when compared to latitude in winter. In their study, Tsiaras and Weinstock (2011) suggest that sufficient UVB radiation for vitamin D_3_ synthesis all year round is sufficient in the region between approximately 35° north latitude and the equator. At higher latitudes, vitamin D_3_ is produced over the winter months. For example, in Rome, Italy (latitude 41.9° North), this is the time of year from February to November. Ten degrees further north in Berlin, Germany (latitude 52.50° North) or Amsterdam, Netherlands (latitude 52.4° North), synthesized from April to October^64^. Holick et al. (2011) conducted a study that revealed important information about the changes in the SZA and how they can significantly affect the synthesis of vitamin D_3_. This is especially true in regions above 33° latitude, where the production of vitamin D_3_ in the skin is very limited or nonexistent during the winter months (from late September to early May). This is because exposure to sunlight is limited because the sun appears at a lower angle. Unfortunately, this causes people in these regions not to get enough exposure to sunlight for vitamin D_3_ synthesis and can ultimately lead to vitamin deficiency^65^.

Seckmeyer et al. (2013), in a study in Hannover, Germany (52.39° North), analyzed the daily value of 1000 IU vitamin D_3_ that could be synthesized according to UV index values for different situations. In the results performed in the vertical position (standing) with 100% body surface exposure area and clear sky, the time taken to reach the adequate level was 39 min on December 21, while this time decreased to 1.1 min on June 21. Moreover, on December 21, this period extends to 35 days in overcast and cloudy weather, making D3 synthesis impossible in Central Europe during winter^66^. Miyauchi et al. (2013) estimated the vitamin D levels by numerical simulation in three cities in Japan, Sapparo (43.04 N), Tsukuba (36.05 N), and Naha (26.12 N). They calculated the time required for skin type III, 600 cm^2^ body area and 5.5 μg (220 IU) production at the Tsukuba location under a cloudless sky. In July, it was 5.9 min at 09:00, 3.5 min at 12:00 and 10.1 min at 15:00. In December; it is 106 min at 09:00, 22.4 min at 12:00 and 271.3 min at 15:00. In addition, the amount of synthesized D_3_ increases proportionally from North to south^67^. Kimlin et al. (2014) studied the latitude-dependent total monthly vitamin D sufficient ultraviolet radiation levels were examined for 0°, 40° and 90°, keeping ozone, altitude and aerosols (pollution) constant. For 0°, February–March and October–November are at their peak, while July is the lowest. For 40°, the peak level occurs in summer (July), while in winter (January), there is almost no vitamin D production. For 90°, there is almost no production for 8 months of the year^68^. Park et al. (2019) the study simulated the amount of vitamin D for 1000 IU (type IV) that can be synthesized depending on the UV index for 6 cities in South Korea between 33.29 and 37.57 N. According to the analysis, the level of vitamin D synthesized decreases proportionally as one moves north. In the summer months (June–August), the time required for synthesis ranges from 12.35 min to 14.10 min, while in the winter months (December to February), the time required for synthesis increases from 50.97 to 99.62 min. Thus, sufficient synthesis is rarely possible by solar UV exposure alone during winter^69^.

Previous studies have shown that vitamin D deficiency is a significant public health issue. The primary and most natural source of vitamin D synthesis is solar radiation. The synthesis of active vitamin D3 is dependent on the latitude at which UVB radiation (280–320 nm) reaches the earth, as well as the seasonal and diurnal position of the sun. This study estimates the time interval for vitamin D synthesis in Antalya and Istanbul Turkey. The study was conducted in three stages. Firstly, daily, monthly, and annual values were determined for SZA angles of 45° and below, which represent the angle at which the UVB wavelength is steepest to the earth's surface. Secondly, the level of vitamin D that can be synthesized for all skin types was estimated based on clear-sky UV radiation. Finally, the study calculated the sun exposure time required for 1000 IU for all skin types. The aim is to minimize the negative effects of the sun, such as photo dermatoses, on individuals who want to benefit from vitamin D synthesis. To sum up, the contribution of this study to the literature is that it provides a UV Index-based model to estimate pre-Vitamin D_3_ synthesis in the Mediterranean basin region and contributes to the knowledge gap regarding the optimal amount of sun exposure required to prevent Vitamin D deficiency.

## Materials and methods

### Solar position calculation

**Figure 2:**
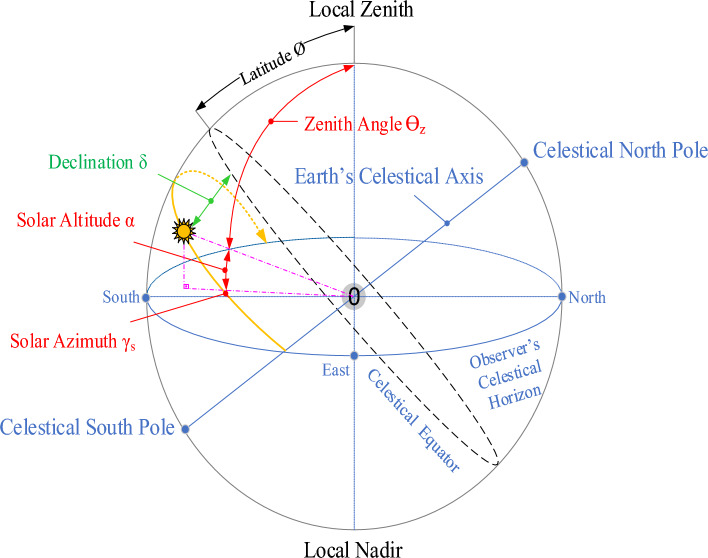
Sun angles positions (daily and yearly).

Using polar coordinates, the latitude, longitude, and altitude of any location on the globe are defined and the movement of the sun in the celestial sphere is analyzed relative to the ground. The position of this movement changes throughout the day and year. Figure 2 shows the solar angles defined by the observer at zero “0”.

In this study, daily (24 h) and yearly (1–365) solar angles of two different geographically located regions were calculated. Among the basic equations used in the calculations, the time correction factor (TC) is shown in Eq. 1^70^. This value is defined as the time since the sun last crossed the meridian and is based on the Earth’s rotation relative to the sun.

1 $$TC=solartime-standarttime=4\left({L}_{st}-{L}_{loc}\right)+E$$

In the equation ${L}_{st}$ latitude for local time zone, ${L}_{loc}$ longitude and E value indicates equation of time (minute) and is expressed by Eq. 2.

2 $$E=229.2\left[0.00075+0.001868{\text{cos}}\left(B\right)-0.032077{{\sin}}\left(B\right)-0.014615{\text{cos}}\left(2B\right)-0.04089{{\sin}}(2B)\right]$$

The B value in Eq. 2 is defined as fractional year and is calculated by Eq. 3. The value n in this equation refers to the number of days in a year (1 ≤ n ≤ 365).

3 $$B=\left(n-1\right) 360/365$$

The declination angle, derived from the 23°27 value of the Earth's axis of rotation with the normal of the orbital plane, is the angle made by the Sun's rays with the equatorial plane and is a function of time only. This angle is zero degrees at the Equinoxes (March 21 and September 23) and reaches its maximum absolute value at the Solstices (June 21 and December 21). Although many equations have been developed in the literature for the calculation of the declination angle value ^71^ most stable (error < 0.0350) is calculated by Eq. 4^72^.

4 $$\updelta =(180/\uppi )(0.006918-0.39912{\text{cos}}B+0.070257{{\sin}}B-0.006758{\text{cos}}2B+0.000907{{\sin}}2B-0.002697{\text{cos}}3B+0.00148{{\sin}}3B)$$

The hour angle ($\omega$) is defined as the angular displacement of the sun east or west of the local meridian. The difference between solar noon and local solar time (LST) is multiplied by the constant number 15 and the solar constant angle is calculated by Eq. 5. This fixed number was obtained by dividing the 360° angle formed during one revolution of the earth around the sun by 24. The hour angle is negative before noon and positive in the afternoon. The LST value in Eq. 5 is calculated by Eq. 6 by considering local time (LT).

5 $$\omega =15\times (LST-12)$$

6 $$LST=LT+\frac{TC}{60}$$

With angles derived as a function of the basic solar angles, it is possible to calculate the solar position. These angles are the Zenith Angle $({\theta \mathrm{z}})$, Sun Elevation Angle $\left({{\alpha }}_{s}\right)$ and Solar Azimuth $(\gamma s)$ happens. The first of these angles is the ${\theta} {\mathrm{z}}$, is the angle that the direct solar radiation direction makes with the normal of the horizontal plane. This angle is used at sunrise and sunset ${\theta} {\mathrm{z}}=90^{\circ}$ whereas in the case where the rays are perpendicular to the earth's surface ${\theta} {\text{z}}=0^{\circ}$ dir. The zenith angle in the horizontal plane (β = 0°) is calculated as in Eq. 7.

7 $$\cos\theta \mathrm{z}={{\sin}}\delta \cdot{{\cos}} \phi {{\cos}}\omega +{{\sin}}\delta {{\sin}} \phi$$

Sun elevation angle ${({\alpha }}_{s})$ is the angle formed between the sun's direct solar radiation and the local horizontal plane at any given moment. It is constantly changing during the day. It also varies according to the latitude of a given location and the day of the year. This angle reaches its maximum value at noon in all seasons. Depending on the declination angle, latitude angle and sundial angle, the following relation is formed.

8 $${{\alpha }}_{s}={{{\sin}}}^{-1}\left[{{sin}}\delta {{\sin}}\phi +{{\cos}}\delta {{\cos}}\phi {{\cos}}\omega \right]$$

The elevation angle at sunrise and sunset is zero, and the sum of the solar elevation angle and the zenith angle (θz + αs = 90°) is ninety degrees.

### Calculated previtamin D_3_ synthesis method

**Figure 3:**
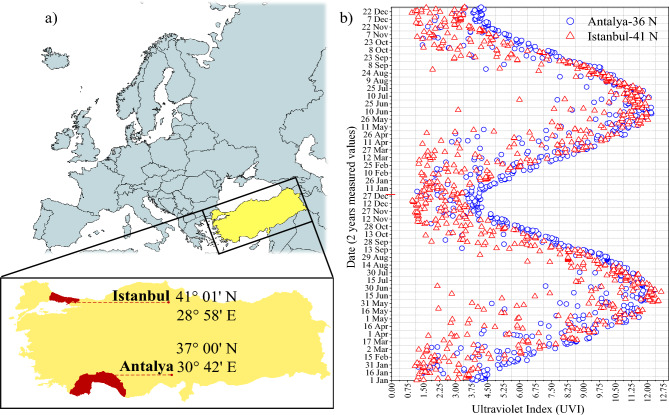
Ultraviolet solar radiation (**a**) measuring location, (**b**) 2 years measured data.

There are both personal and environmental factors involved in the synthesis of vitamin D. These include latitude, seasons, altitude, time of day, air pollution, cloud cover, use of protective cream, melanin content in the skin, age, weight, and the amount of clothing covering the body. It has been described in the literature as the “Hollick Rule” that exposure of approximately 1/4 of the body (BSA), 1/4 minimum erythemal dose (MED) is equivalent to an oral vitamin D_3_ intake of approximately 1000 International Units (IU)^73^. UV-Index values used in the synthesis of vitamin D in this study were taken by the Meteosat satellite (Heliosat-3) within the Helioclim-3 program provided by Mines Paristech/Armines/ Transvalor S.A^74^.This satellite system processes UVA, UVB and UV total values into the database daily and monthly for 2004–2005. The geographical coverage corresponds to the Meteosat satellite field of view, i.e. covers Europe, Africa, Atlantic Ocean, and Middle East. Spatial resolution is 3 km at Nadir and is increasing as soon as we get away from this point^75,76^. The results from this satellite-based process the data for 2 years (730/days) with daily average UV index values for Antalya and Istanbul are shown in Fig. 3 and Table 1. The UV index data shown in Fig. 3 was obtained from “SoDa” and edited in Matlab software^77^. Antalya is located at 37 N 00 latitude and 30 E 42 longitude (Turkey) on the coast of the Mediterranean Sea at an average altitude of 39 m and shows Hot-summer Mediterranean (Csa) climate characteristics. Istanbul is located at 41 N 01 latitude and 28 E 58 longitudes (Turkey) on the coast of the Marmara Sea at an average altitude of 40 m and shows the characteristics of the Hot-summer Mediterranean (Csa) climatealtitude affects the total amount of UV^78^. At relatively higher altitudes, aerosols, tropospheric ozone, and clouds are less abundant than at sea level, leading to a decrease in the amount of UV absorbed. For every 1000 m of altitude effect, the daily midday mean of global UV radiation increases by about 10.7%. In addition, it is important to note that the UV Index varies depending on the surface’s albedo. Surfaces such as grass, soil, or water reflect less than 10% of incoming UV radiation, while dry beach sand reflects about 15% and sea foam about 25%. Fresh snow is an especially effective reflector, nearly doubling a Person’s UV exposure. This study used an average annual albedo of 0.30 (soil-dry). For this purpose, the provinces selected to standardize the calculations are located at sea level^79,80^.

**Table 1:** Monthly change of meteorological data.

Months	UV index (mW/m^2^)		Total ozone (DU)		Solar radiation (Wh/m^2^-day)		Temperature (°C)		Sunshine duration (h)	
Region	Antalya	Istanbul	Antalya	Istanbul	Antalya	Istanbul	Antalya	Istanbul	Antalya	Istanbul
January	3.6	2.5	320	331	2058	2000	10	6.7	5.1	3.46
February	4.9	3.4	329	350	2804	2570	10.7	6.9	5.8	4.43
March	6.8	5.2	341	367	4045	4200	12.9	8.4	6.7	5.32
April	8.4	7.4	337	354	5183	5280	16.4	12.8	7.9	6.85
May	10.3	9.2	336	345	6390	6300	20.6	17.6	9.7	8.61
June	11.1	10.6	309	332	6610	6790	25.3	22.2	11.3	10.51
July	11.0	10.8	292	308	6701	6790	28.4	24.6	11.7	11.17
August	10.2	9.5	286	296	5957	6070	28.4	24.6	11.2	10.14
September	8.0	6.8	286	299	4697	5090	25.2	21.1	9.7	7.83
October	5.9	4.4	290	287	3293	3740	20.5	16.6	7.8	5.22
November	4.2	2.7	292	302	2370	2370	15.4	12.5	6.3	3.85
December	3.7	2.3	298	305	1926	1800	11.6	8.9	4.8	2.96
Average	7.34	6.23	310	323	4336	4417	19	15	8	7

This study calculated the UV index value for the synthesis of vitamin D. Previous research has shown that there is no significant difference between the satellite-based measurements and ground-based measurements in mid-latitude Europe for clear-sky model^81,82^. Cloud cover, usually measured in octas, is not the most reliable way to measure the reduction in UV radiation caused by clouds and the resulting fluctuations^83^. For this study, calculations have been made for the period when the solar zenith angle (SZA) is at its lowest, ensuring the least attenuation rate for solar radiation. The annual average total ozone value (DU) of the two regions used in the calculations varied between 310 and 323. By taking into account all these factors, the study provides reliable calculations for vitamin D synthesis.

Equation 9 shows the equation that formulates Erythema-weighted. The UV index in this equation is a dimensionless parameter and can be obtained most clearly by dividing erythema-weighted (Ery) by 0.25 Wm^−2^. Also in the equation ${H}_{\lambda }$ solar radiation incident on the horizontal surface (mW/m^2^/nm), ${w}_{\lambda }$ the spectrum of the erythemal effect and the weight of the wavelength range of the radiation are shown in Table 2^84^.

**Table 2:** Erythemal effect spectrum ranges.

Wavelength	Erythemal weighting function
$250 nm<\lambda \le 298 nm$	${w}_{\lambda }=1$
$298 nm<\lambda \le 328 nm$	${w}_{\lambda }={10}^{0.094(298-\lambda/nm)}$
$328 nm<\lambda \le 400 nm$	${w}_{\lambda }={10}^{0.015(139-\lambda /nm)}$
$400 nm<\lambda$	${w}_{\lambda }=0$

9 $${UV}_{Ery}=k{\int }_{252}^{330}{{{H}_{\lambda }w}_{\lambda }d}_{\lambda }=k\left[\frac{{m}^{2}}{W}\right].EUV\left[\frac{W}{{m}^{2}}\right]$$

The minimal erythema dose (MED), UV index value and body surface area (BSA) are functions of vitamin D synthesis. Equation 10 shows the linear synthesis model according to Hollick’s rule.

10 $${UV}_{d}=\frac{MED}{4} x\frac{0.25}{BSA} x \frac{{D}_{vit}}{1000}$$

The minimal erythema dose (MED-J/m^2^) varies depending on the skin type and is shown in Table 3.Sun protection factor (SPF) and tanning values, which prevent UVB radiation from being effective, are considered ineffective in this study. This is to disable more complex parameters. One standard erythema dose (SED) is equal to 100 Jm^−2^ in weight. Thus, the SED per hour can be found by multiplying the erythema-weighted value by 36 (SED per hour xm^2^ W^−1^)^32,59^. Body surface area is calculated as the surface area exposed to UV rays. The amount of synthesis increases as the area of skin exposed to the sun (BSA), which is effective in vitamin D synthesis, increases. In this study, the body surface areawas fixed at 25% according to the “Hollick rule”^85^.

**Table 3:** Minimal Erythemedose and skin types.

Skin type	I	II	III	IV	V	VI
1 MED (J/m^2^Erthema)	200	250	300	450	600	1000
Tanning ability	Always burns, does not tan	Burns easily, tans poorly	Tans after initial burn	Burns minimally, tans easily	Rarely burns, tans darkly easily	Never burns, always tans darkly
Skin color	Light	Light	Medium	Medium/dark	Dark	Black
Hair color	Red/blond	Red/blond/Light brown	Chestnut/dark blond/ lightandmedium brown	Medium anddark brown/black	Dark brown/black	Black
Eye color	Blue/green/grey	Blue/green/grey/hazel	Brown/blue/green/grey/hazel	Hazel/medium anddark brown/black	Dark brown/black	Black

The UV_d_ value is the time-dependent UV exposure shown in Eq. 11. The value “ker” in the equation is a constant coefficient equal to 40 (m^2^/w). The “t” value indicates time, and its unit is second. In the first stage of the calculations, the radiation exposure time was assumed constant at 10 min ^26^. The time required for 1000 IU/day was then calculated.

11 $${UV}_{d}=\frac{UVI x t}{{k}_{er}}$$

Hollick’s rule calibrated the differences between the values obtained from active sunlight and the experimental values obtained from UVB lamps^86^. As a result of this process, UV exposure estimated based on solar conditions outperforms the effectiveness of pre-vitamin D_3_ UV exposure using fluorescent lamps by 32%. Therefore, a new form using the correction factor is written in Eq. 12^87^.

12 $${UV}_{d}=\frac{1}{1.32} x \frac{1}{16000}x\frac{MED x {D}_{vit}}{BSA}$$

When Eqs. 11 and 12 are combined, the calculation of vitamin D level according to Hollick’s rule D_vit_ synthesis model is shown in Eq. 13^88^.

13 $${D}_{vit}=21120 \frac{UVI x t x BSA}{{k}_{er} x MED}$$

### Ethical approval

Since (in-vitro) theorical analyses were performed in this study, no ethics committee approval document is required. Additionally, no live animals or individuals were used in the study. This material is the authors’ own original work, which has not been previously published elsewhere. The human participants are not involved in the study/the data used is publicly available.

## Results and discussion

### Solar elevation angle and UV index

**Figure 4:**
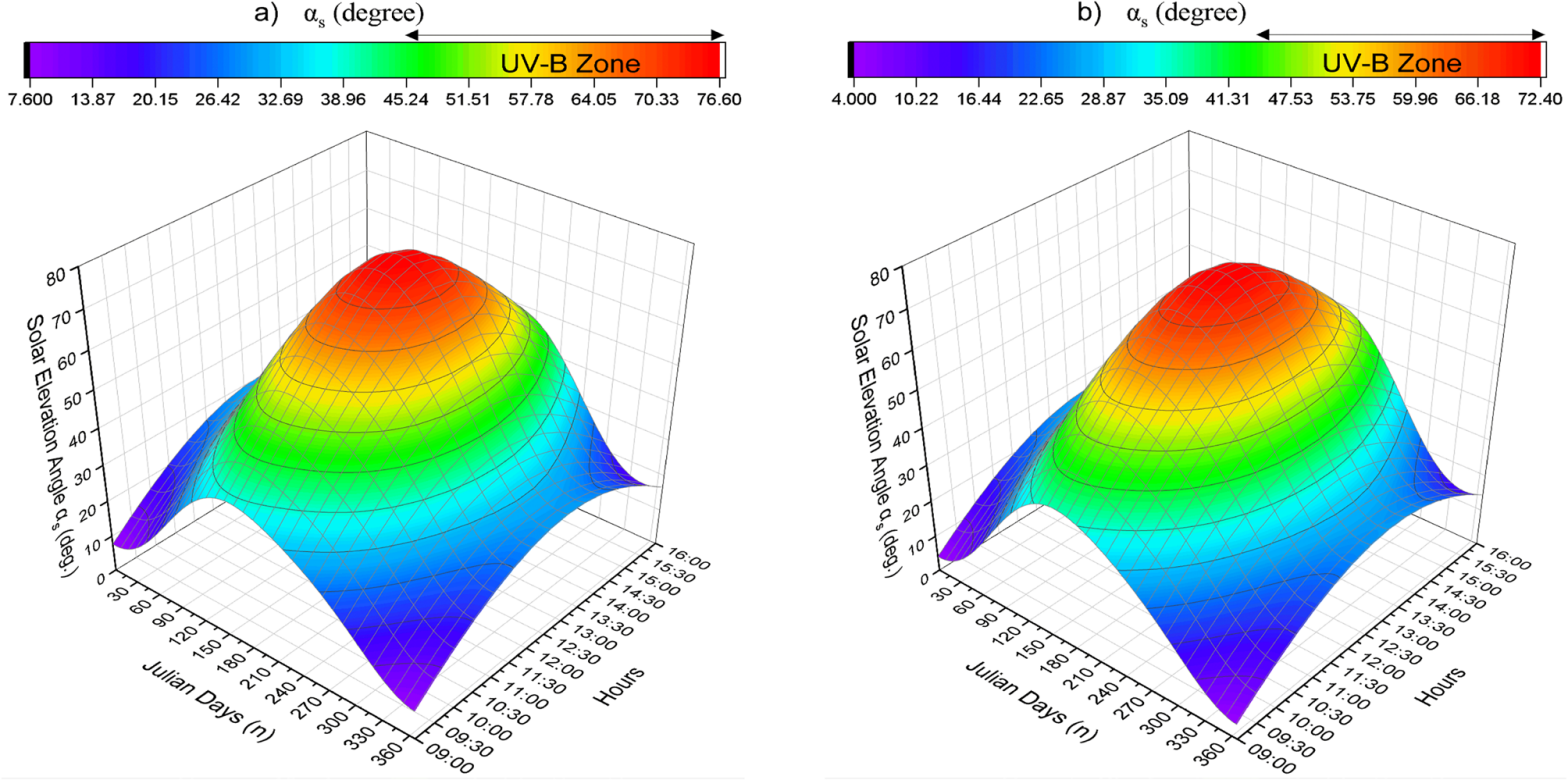
UV-B zone (**a**) Antalya (**b**) Istanbul.

The change in solar elevation angle (deg) during the year is shown in Fig. 4 in hourly (09:00–16:00). In Antalya and Istanbul, the solar elevation angle value for 09:00 and 09:30 does not exceed 45 degrees. The amount of UVB required for adequate vitamin D synthesis occurs at high α_s_ > 45°. The wavelengths required for vitamin D synthesis were analyzed at the beginning and end of the year. In this case for Antalya and Istanbul respectively; 10:00 starts at 29/April (118 JD)-11/May (131 JD) and ends at 05/August (217 JD)-20/July (201 JD). For 10:30, it starts on 08/April (98 JD)—19/April (109 JD) and ends on 05/September (248 JD)—21/August (233 JD). For 11:00, it starts at 25/March (84 JD)-05/April (95 JD) and ends at 23/ September (266 JD)-09/ September (252). For 11:30, it starts at 15/March (74 JD) -26/March (85 JD) and ends at 04/October (277 JD) -21/September (264 JD). For 12:00, 07/March (66 JD) -19/March (78) starts and 15/October (283 JD) -28/September (271) ends. For 12:30 starts at 02/March (61 JD)-13/March (72 JD) and ends at 22/ October (285 JD)-02/September (275 JD). For 13:00 starts at 28/February (59 JD)-10/March (69 JD) and ends at 14/October (287 JD)-03/September (276 JD). For 13:30 starts at 01/March (60 JD)-12 March (71 JD) and ends at 10/October (283 JD)-30/October (273 JD). For 14:00 starts at 04/March (63 JD)-14/March (73 JD) and ends at 04/October (277 JD)-26/September (269 JD). For 14:30 starts at 11/March (70 JD)-20/March (79 JD) and ends at 24/September (267 JD)-19/September (262 JD). For 15:00 starts at 22/March (81 JD)-30/March (89 JD) and ends at 15/September (258 JD)-10/September (253 JD). For 15:30 starts at 26/April (100 JD)-14/April (104 JD) and ends at 1/September (244 JD)-28/August (240 JD). For 16:00 starts at 26/May (JD 131)-11/May (JD 131) and ends at 11/ August (JD 223)-09 August (JD 221).

**Figure 5:**
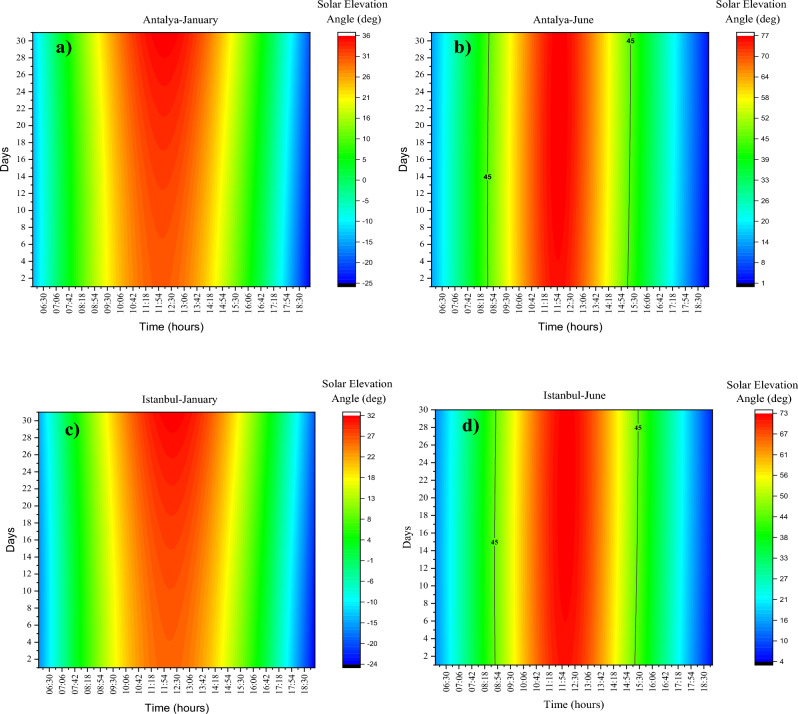
Solar elevation angle (deg) (**a**) Antalya-January (**b**) Antalya-June (**c**) Istanbul-January (**d**) Istanbul-June.

In all months, the highest values are observed between 11:30 and 13:30 at noon. In the months of the analyses, the UVB time frame (αs < 45°) is not observed at all in January, November, and December and partially in February and October. From the Capricorn equinox (December 21) a positive correlation is observed between the increases in the diurnal solar elevation angle until the crab equinox (June 21). For the northern hemisphere, June 21 is the solstice and the time when the sun's rays are at their steepest. As can be seen in Fig. 5, this value peaks on June 21 and falls to its lowest level on December 21. Active D_3_ synthesis does not occur for cities in December. According to the average values of June (162 n), it occurs between 08:42 and 15:18 and between 08:48 and 15:07for Antalya and Istanbul, respectively. The longest daytime interval of D_3_ synthesis during the year is 396 min and 376 min for Antalya and Istanbul, respectively.

### Vitamin D_3_synthesis levels

**Figure 6:**
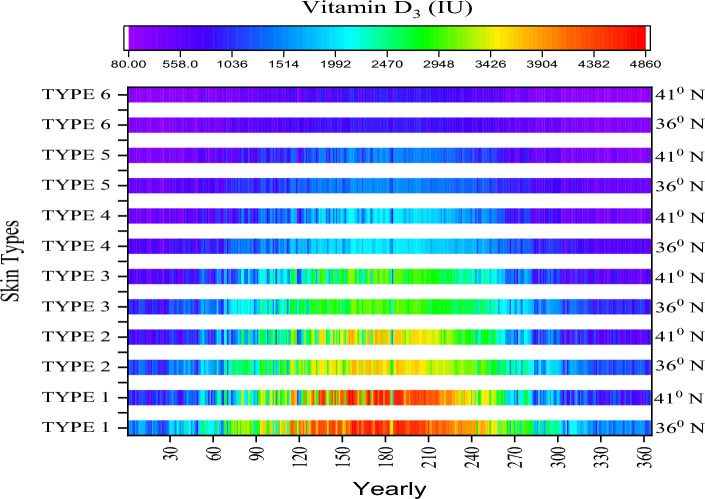
Yearly vitamin D_3_ production rates.

In the final stage of vitamin D synthesis, the parameters in Eq. 8 are skin type, body surface area;exposure time and UV index values. In Fig. 6, the amount of vitamin D_3_ synthesis is determined from meteorological data measured in Antalya (37° N) and Istanbul (41° N) for six cases (I–VI) in different skin type. In the calculations, the sun exposure time was assumed to be 10 min and the body exposure area (BEA) was 25%. Depending on the latitude, the amount of D_3_ synthesized varies. The main reason for this is that the UV index value increases proportionally as UV rays travel the shortest distance to reach the earth as they approach the equator (0° N) from the north pole (90° N). As a result, the amount of vitamin D_3_ synthesized in Antalya for the whole year (1–365) is 17.6% higher for all skin types compared to Istanbul.

In the summer months only (152–243), this rate is 5.1%. When UVB levels are sufficient, the amount of D_3_ synthesized is highest in skin type I and decreases as you go to type VI. The main reason for this is the variability of 1 MED (J/m^2^ Erythema). As a result of these values, the period with the highest synthesis is in the summer months, while the lowest period is in the fall and spring seasons. However, since the level of UV exposure in the summer season is at an extreme level, individuals should be conscious and use protection (sunscreen) when necessary. There is no international consensus on the safe upper level for vitamin D supplementation. Numerous institutions and scientific organizations have developed recommendations for optimal serum vitamin D concentrations. The level of vitamin D needed varies depending on age, body weight, chronic disease status and ethnicity.

**Figure 7:**
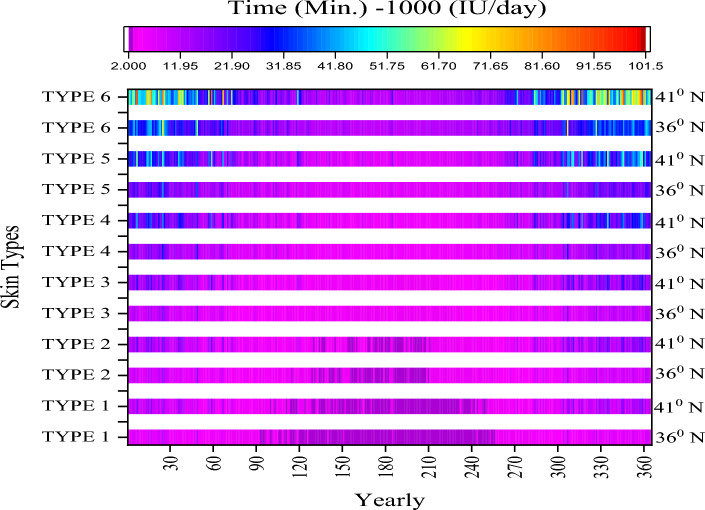
Optimum sun exposure time.

The National Institutes of Health (NIH) sets a lower limit of 400 IU/day (10 mcg) for infants up to 1 year of age and an upper limit of 800 IU/day (20 mcg) for people over 71 years of age. The maximum tolerated dose is 1000 IU/day (25 mcg) in infants up to 1 year of age, while the upper limit is 4000 IU/day (100 mcg) in people over 71 years of age^89^. The upper daily limit recommended by the Endocrine Society is 10,000 IU/day, while the Institute of Medicine (IOM) recommends staying below 4000 IU/day^90^. The recommended daily intake of vitamin D for a Person with normal vitamin D levels is 1000 IU per day, according to the guidelines established by the Institute of Medicine. If this amount is taken regularly, it is expected to result in a 10 ng/ml increase in blood vitamin D levels after a period of 3–4 months. However, it’s worth noting that this is a general estimate and individual responses to vitamin D supplementation may vary depending on various factors such as age, gender, skin color, sun exposure, and underlying health conditions. Therefore, it’s important to consult a healthcare professional before beginning a vitamin D supplement regimen to determine the appropriate dosage and ensure that it’s safe for your specific needs. Furthermore, it's advisable to get regular blood tests to monitor your vitamin D levels and adjust the dosage if necessary^1^. In support of this study, Çağlayan et al. (2022) the change in blood vitamin D (25-OHD) levels in Turkey are seasonal. Thus, there is a positive correlation between increased sun exposure and 25-OHD level. However, this rate was observed less in women than in men. In the study conducted on 1,634,618 people, the greatest level of variation in the measured values (from winter to autumn) is around 5.1 ng/ml in men and 3.8 ng/ml in women^91^. In this study, the predicted average cumulative increase amount (899–2095 IU) for type III from winter to autumn is 11.95 ng/ml for Antalya and Istanbul. According to the vitamin D researcher “Hollick’s rule”, a minimum erythemal dose (1 MED) of approximately 25% of the body (lower part of the hands, arms and feet) is equivalent to an oral intake of approximately 4000 IU of vitamin D^85^. Figure 7 shows the amount of sun exposure (minutes) by skin type for a daily maintenance dose of 1000 IU/day (25 mcg) determined according to this rule.

The overall average values are 4.2 min, 5.2 min, 6.3 min, 9.4 min, 12.5 min, 20.9 min for Antalya in Skin type I–II–III–IV–V and IV, respectively. For Istanbul, Skin type I–II–III–IV–V and IV are 5.9 min, 7.4 min, 8.9 min, 13.3 min, 17.8 min, and 29.6 min respectively. The mean values for spring are 3.2 min, 4 min, 4.8 min, 7.2 min, 9.6 min, and 16 min in Skin type I–II–III–IV–V and IV for Antalya, respectively. For Istanbul, Skin type I–II–III–IV–V and IV are 4 min, 5.1 min, 6.1 min, 9.1 min, 12.1 min, and 20.2 min respectively. The average values for summer are 2.3 min, 2.9 min, 3.5 min, 5.3 min, 7 min, and 11.7 min in Skin type I–II–III–IV–V and IV for Antalya, respectively. For Istanbul, Skin type I–II–III–IV–V and IV are 2.5 min, 3.1 min, 3.8 min, 5.7 min, 7.5 min, and 12.6 min respectively. Unlike the specified time value, to reach 2000 IU; for 2 times the duration or 50% BSA also 4000 IU; it is possible to get 4 times the duration or 100% BSA. In this way, it is possible to reach 30–40 ng/mL levels from low levels when needed. It is anticipated that the times indicated may be sufficient for a daily maintenance dose in the absence of sunscreen (SPF) and tanning (0%). The values obtained from this study were compared with two different calculation methods. First of all, the amount of D_3_ synthesis calculated depending on the UV exposure in the horizontal plane used in this study was compared to the work of McKenzie et al.^92^. Parameters accepted in the calculations, skin type: II, CIE action spectrum as a function of ozone and SZA, body exposure area: 100%, D_3_ dose: 1000 IU, and assumption variable according to UV index is input. In this situation, the maintenance dose duration for Antalya and Istanbul is shown in Table 4. The difference between the two results is very small and is at an acceptable level.

**Table 4:** Validation of calculation times values for McKenzie et al. (2009).

Methods	Antalya		Istanbul	
	Calculated	McKenzie et al. (2009)	Calculated	McKenzie et al. (2009)
March	1.2	1.6	1.5	2.3
April	0.9	1.3	1.1	1.5
May	0.8	1.0	0.9	1.1
June	0.7	0.9	0.7	0.9
July	0.7	0.9	0.7	0.9
August	0.8	1.0	0.8	1.1
September	1.0	1.3	1.2	1.6

**Figure 8:**
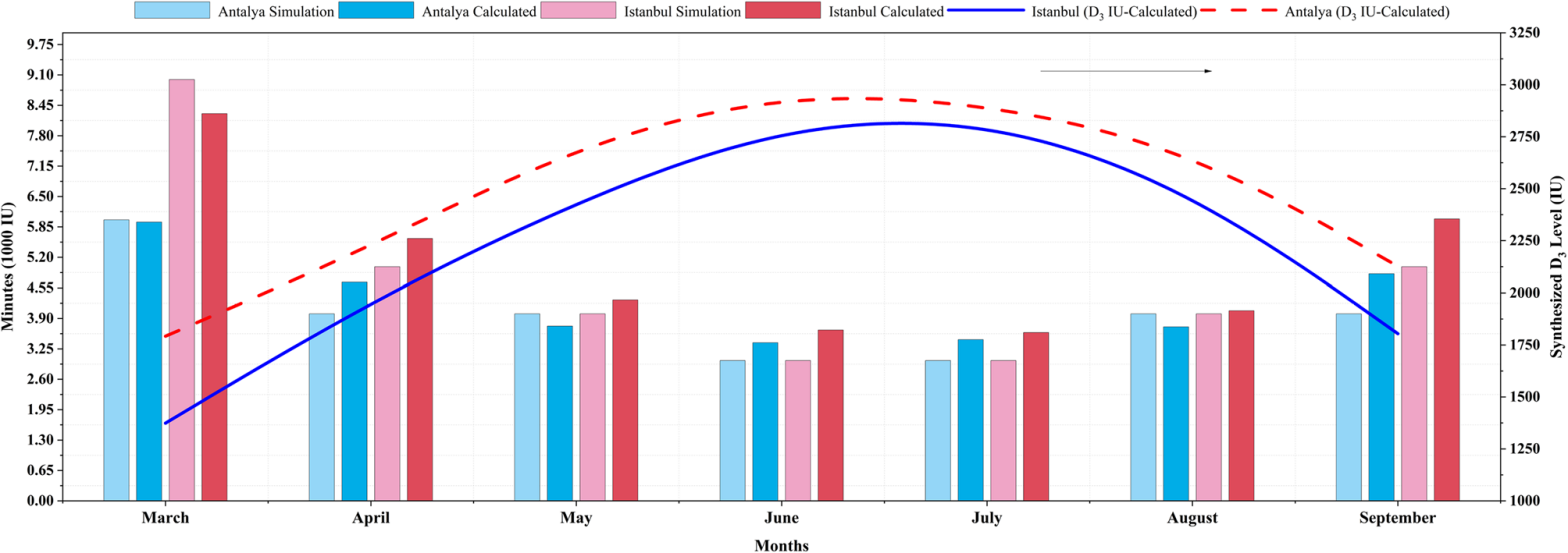
Validation of calculation values for FastRT simulation.

The second method is, the data obtained from the study were provided with the “FastRT” simulation program. This simulation calculates UV doses, UV indices, and irradiances as small as 0.05 nm in the spectral range from 290 to 400 nm^62,93^. Parameters accepted in the calculations, popular Turkish skin type: III, total ozone column: change by monthly (Table 1), surface altitude: 0.10 km, surface albedo: 0.30 (soil-dry), body exposure area: 25%, D_3_ dose: 1000 IU, timing of exposure: around midday and clear-sky assumption variable according to UV index is input. The calculated and simulated daily sun exposure times for Antalya and Istanbul are shown in Fig. 8. The calculated values of the cities in the figure are the information obtained from this study and the simulated values are obtained from the program.

When the calculated result is compared with the simulation, the values in the active D_3_ season (spring, summer, and fall) were very close. The approximation error percentages (e %) for Antalya during March–October were recorded as 0.84, 14.35, 8.11, 11.24, 11.76, 7.53, and 17.36, respectively. Comparatively, those for Istanbul were 8.83, 10.71, 6.76, 17.81, 16.67, 1.48, and 16.94. Overall, these values are within acceptable levels for both cities.

The differences in the amount of Vitamin D_3_ synthesized in the maintenance dose of skin Type III between the provinces of Antalya and Istanbul were measured by examining the intra-year changes. Monthly, the percentages for March, April, May, June, July, August, September, and October were 23%, 11%, 10%, 5%, 2%, 7%, and 15%, respectively. Additionally, the D_3_ (IU) levels for each (1°) latitude degree were measured for March, April, May, June, July, August, September, and October, and the respective differences in values were 105, 64, 68, 39, 18, 48 and 80. When comparing the provinces of Antalya and Istanbul, Antalya, which is located closer to the equator, exhibited a higher total amount of D_3_ synthesized throughout the year with less fluctuation between the summer and winter months.

## Conclusions and recommendations

In this study, the synthesis of vitamin D was analyzed from solar radiation throughout the year for Antalya at 37° and Istanbul at 41° in the northern hemisphere. In the first stage of the study, the annual and daily active period of UVB radiation was determined depending on the solar elevation angle. Thus, the synthesis of 7- dehydrocholesterol (7-DHC) is activated by the ultraviolet effect of sunlight through the skin. In the second stage, possible D3 synthesis values were calculated from satellite-based UV index measurement data. Overall, the results are in the listings;

- Earth’s active UVB time zone starts after 10:00 and continues until 16:00. The peak period is in the summer months.
- The longest period for UVB is at 12:30 pm. The period of active D_3_ synthesis lasts from the beginning of March until the third week of October.
- The UVB effect of radiation is absent in January, November, and December. In February and October, it is almost nonexistent.
- Since the UV index value in the summer season is at an extreme level, it is recommended that individuals with sensitive skin who may be exposed to the sun for a long time should use protection. In addition, UVI > 11 in the middle of the day should give individuals more attention to disease risks.
- When D_3_ Synthesis is analyzed, the average estimated times (minutes) for 1000 IU/day are 5.05 for type I, 6.3 for type II, 7.6 for type III, 11.35 for type IV, 15.15 for type V and 25.25 for type VI.
- For every degree of latitude from the equator to the poles, the average amount of D_3_ decreases by 105 IU in summer and 237 IU in spring.
- The amount of D_3_ synthesis (IU) increases by 10.7% at 1000 m above sea level and by 15% at the coast. Similarly, the time required for the return dose (min) decreases.
- The values obtained from the analysis of the central Mediterranean region can be applied to other coastal areas around the world that share similar latitudes and climatic conditions (Csa). This region includes Malaga and Barcelona in Spain, Lisbon in Portugal, Naples and Bari in Italy, Athens in Greece, Algiers in Algeria, and Tunis in Tunisia.
- Sunlight has benefits, but extended exposure can cause skin diseases. This study analyzed the ideal insolation interval between 37 and 41 degrees north latitude. Sunbathing 3–4 times a week for a minimum of 12 and a maximum of 34 min (depending on skin type) without protective cream during active UVB time zones in spring, summer, and fall is enough to meet vitamin D needs. However, exposure to winter sun alone is insufficient.

## Data Availability

The data can be obtained from the Fisrt author and corresponding author on reasonable account.
